# MiR-125a-5p Decreases the Sensitivity of Treg cells Toward IL-6-Mediated Conversion by Inhibiting IL-6R and STAT3 Expression

**DOI:** 10.1038/srep14615

**Published:** 2015-10-01

**Authors:** Dan Li, Chao Kong, Andy Tsun, Chen Chen, Huihui Song, Guochao Shi, Wen Pan, Dai Dai, Nan Shen, Bin Li

**Affiliations:** 1 Key Laboratory of Molecular Virology & Immunology, Unit of Molecular Immunology, Institut Pasteur of Shanghai, Shanghai Institutes for Biological Sciences, Chinese Academy of Sciences, Shanghai 200031, China; 2 Shanghai Key Laboratory of Bio-energy Crops, College of Life Science, Shanghai University, Shanghai 200444, China; 3 Department of Pulmonary Medicine, Rui Jin Hospital, School of Medicine, Shanghai Jiao Tong University, Shanghai 200025, China; 4 State Key Laboratory of Oncogenes and Related Genes, Shanghai Cancer Institute, Renji Hospital, Shanghai Jiaotong University School of Medicine, Shanghai, China; 5 Joint Molecular Rheumatology Laboratory of the Institute of Health Sciences and Shanghai Renji Hospital, Shanghai Institutes for Biological Sciences, Chinese Academy of Sciences, and Shanghai Jiaotong University School of Medicine, Shanghai 200025, China; 6 Division of Rheumatology and the Center for Autoimmune Genomics and Etiology (CAGE), Cincinnati Children’s Hospital Medical Center, Cincinnati, OH 45229, USA; 7 Department of Genetics, Yale University School of Medicine, New Haven, CT 06520, USA

## Abstract

The transcription factor FOXP3 is essential for the differentiation and function of regulatory T cells (Treg). It is established that the transcription factor GATA-3 is induced in Treg cells under inflammatory conditions. GATA-3 stabilizes FOXP3 levels to avoid the differentiation of Treg cells into inflammatory-like T cells. The IL-6 signal pathway influences the sensitivity of Treg cells towards instability. The mechanism of GATA-3 in regulating FOXP3 and its relation to the IL-6 pathway remains unclear. Here we report how miR-125a-5p plays an important role in regulating the conversion of Treg cells by IL-6. miR-125a-5p expression is low in Treg cells under steady state conditions and can be induced by GATA-3 to inhibit the expression of IL-6R and STAT3. This finding reveals a GATA3/miR-125a-5p/IL-6R and STAT3/FOXP3 regulatory pathway, which determines how Treg cells respond to inflammatory IL-6-rich conditions.

Treg cells maintain the balance of immune self-tolerance and homeostasis via limiting aberrant or excessive inflammation^1^,^2^. Treg cells are not terminally differentiated cells, as they can lose the expression of FOXP3 and become pro-inflammatory cells when induced by certain cytokines^3^,^4^,^5^. IL-6 is one of the most likely candidates to induce FOXP3 downregulation as it plays a critical role in determining the balance between Th17 and Treg differentiation^6^. In year 2003, Pasare and Medzhitov reported that Toll-like receptor-induced IL-6 expression led to the loss of Treg cell function^7^. More recently, activated Treg cells were found to differentiate into Th17 cells in the presence of IL-6 and in the absence of exogenous TGF-β^8^. IL-6, together with IL-1, may induce downregulation of Foxp3 in a signal transducer and activator of transcription 3 (STAT3)-dependent pathway^9^. *In vivo* experiments have also shown that in autoimmune arthritis FOXP3+ Treg cells lose FOXP3 expression and undergo conversion into Th17 cells. This process was found dependent on IL-6^10^. It has been reported that IL-6 downregulates FOXP3 mRNA expression via epigenetic changes^11^. Furthermore, our laboratory has shown that IL-6 and TGF-β treatment can downregulate FOXP3 by promoting FOXP3 protein degradation *in vitro*^12^. However, how the IL-6 signal pathway is regulated in Treg cells remains unclear.

MicroRNAs (miRNAs) have emerged as important regulators in many physiological and pathological processes including development, differentiation, metabolism, immunity, cell proliferation and apoptosis^13^,^14^. miRNAs are small non-coding RNAs, which repress translation or cleave messenger RNAs (mRNAs) via binding to the coding sequence (CDS) regions or the 3′ untranslated regions (3′UTR) of target genes. For instance, miR-146a has been reported as indispensable for Treg-mediated suppression through regulating the IFNγ response by targeting STAT1^15^. FOXP3 directly induces miR-155, and is responsible for the survival of Treg cells through repression of suppressor of cytokine signaling 1 (SOCS1)^16^,^17^. All these findings suggest that miRNAs play important roles in the function of Treg cells.

In this study, we performed a miRNA microarray and Real-Time-PCR analysis on a human Treg-like cell line to correlate miRNA expression with FOXP3 expression. We identified that miR-125a-5p, a GATA3-inducible miRNA, targets interleukin 6 receptor (IL-6R) and STAT3 transcripts. We propose a regulatory mechanism by which miR-125a-5p reduces the sensitivity of Treg cells toward IL-6 conversion.

## Results

### mir-125a-5p expression is regulated in human T cell lines and T subsets

**Figure 1:**
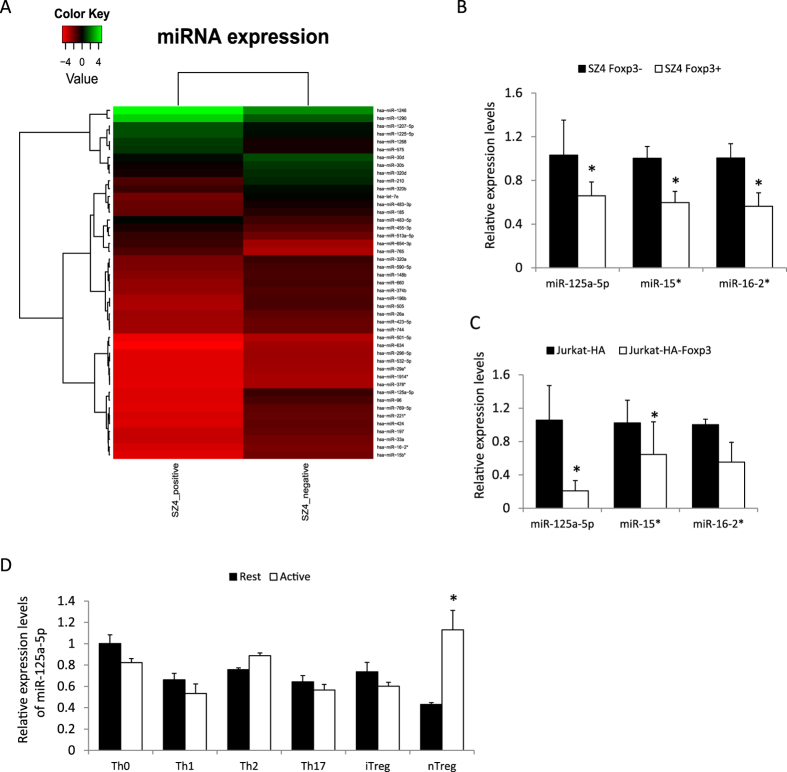
Expression profile of miRNAs in human T cell lines and T cell subsets. (**A**) Microarray data of miRNAs in FOXP3+ and FOXP3− SZ4 cell lines. The clusters of all the miRNAs that had more than twofold change are shown in this figure. (**B,C**) Detection of mature miR-125a-5p, mir-15* and miR-16-2* by real-time PCR in SZ4 cell line (**B**) and Jurkat cell line (**C**). (**D**) The expression levels of miR-125a-5p in different T cell subsets detected by Real-Time PCR. U6 RNA was used for the normalization control. Each data point was obtained in triplicate. *p < 0.01 (t-test).

FOXP3 is the master transcription factor in Treg cells, which regulates the function of Treg cells via modulating gene transcription. We were interested in knowing whether any miRNAs were related to FOXP3 expression. The SZ-4 cell line was originally identified in a Sezary disease patient and expresses FOXP3^18^. This cell line has been used to investigate Sezary Syndrome and the regulation of genes in T cells^19^. We purified RNA from the FOXP3-positive SZ-4 cell line and FOXP3-negative SZ-4 cell line (derived from subcloning) and carried out miRNA microarray to compare expression levels of miRNA. The clusters of miRNAs that had more than two-fold change in expression were rendered into a heat-map diagram (Fig. 1A). miR-125-5p was one of the most downregulated miRNAs revealed by the microarray data. We then used Real-Time-PCR to confirm the expression levels of several miRNAs in the SZ-4 (Fig. 1B) and Jurkat (Fig. 1C) cell lines. All of these results indicated that miR-125a-5p is downregulated in FOXP3-positive T cells. We then checked the expression levels of miR-125a-5p in primary Th0, Th1, Th2, Th17, iTreg and nTreg cells. The expression of miR-125a-5p was found lower in resting nTreg cells compared with effector T cells, but could be upregulated after TCR activation (Fig. 1D).

### GATA3 induces the expression of miR-125a-5p in Treg cells

**Figure 2:**
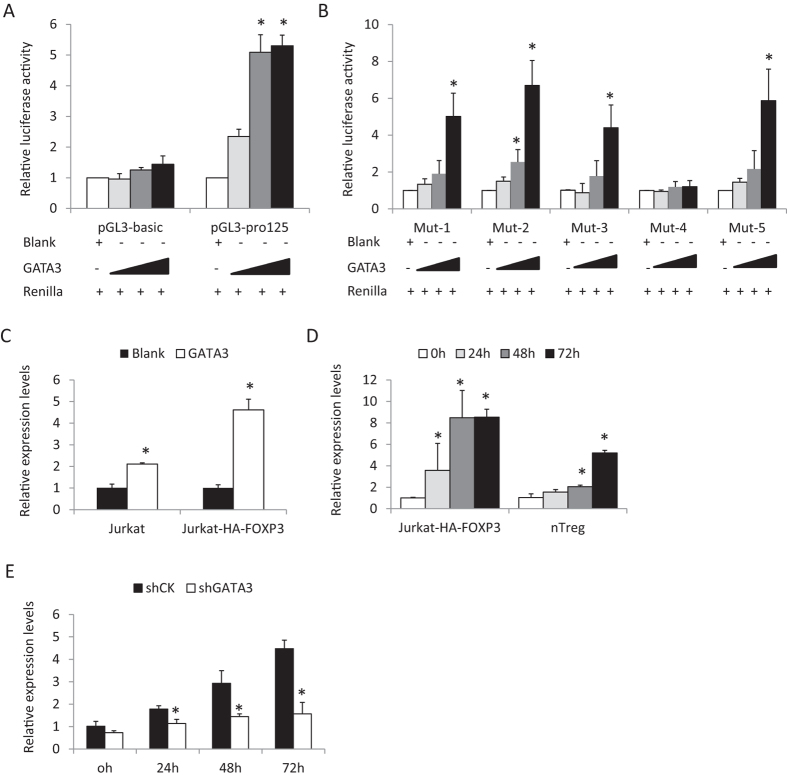
miR-125a-5p is upregulated by GATA3. (**A**) Luciferase assay of the promoter of miR-125a-5p in 293T cells. (**B**) Luciferase activity of reporter vectors with mutations in the promoter of miR-125a-5p in 293T cells. The luciferase activities were detected 48 h after transfection. (**C**) The expression levels of miR-125a-5p were detected by Real-Time PCR in Jurkat and Jurkat-FOXP3 cells after GATA3 overexpression. (**D**) After TCR activation, the Real-Time PCR measurements were measured for the expression levels of miR-125a-5p in Jurkat-FOXP3 cells and primary human nTreg cells at different time-points. (**E**) The expression of miR-125a-5p in GATA3-depleted Treg cells after activation. *p < 0.01 (t-test).

In order to know whether the expression of miR-125a-5p is regulated by FOXP3, we analyzed the promoter region of miR-125a-5p for transcription factor binding DNA epitopes. There were no predicted binding sites for FOXP3, but rather those for GATA3 binding were found (Supplemental Sequence 1). We then cloned the promoter region of the gene transcribing miR-125a-5p into the pGL3-basic vector and carried out a luciferase assay in 293T cells (Fig. 2A). The data indicated that the activity of the miR-125a-5p promoter could be induced by GATA3 in a dose dependent manner. Site-directed mutations suggested that site four was the most important binding site of GATA3 as luciferase activity after mutation of the fourth site showed no change of luciferase activity with different levels of PIP-Myc-GATA3 (Fig. 2B).

We then examined whether GATA3 could upregulate miR-125a-5p levels. GATA3 was overexpressed in Jurkat and Jurkat-HA-FOXP3 cells by electroporation and we found the expression level of miR-125a-5p was upregulated (Fig. 2C). Endogenous GATA3 was then induced by anti-CD3/CD28 antibodies in Jurkat-HA-FOXP3 cells and primary human nTreg cells. Protein levels of GATA3 was tested via Western blotting (Supplemental Fig. 1A) or flow cytometry (Supplemental Fig. 1B) and the expression level of miR-125a-5p though Real-Time PCR (Fig. 2D). These results show that miR-125a-5p could be upregulated and this correlated with the endogenous induction of GATA3 expression.

We then depleted GATA3 via lentivirus transduction in nTreg cells followed by anti-CD3/CD28 activation, and detected GATA3 protein and miR-125a-5p levels. The results show that the upregulation of miR-125a-5p in activated nTreg cells was dependent on endogenous GATA3 expression (Fig. 2E & Supplemental Fig. 1C). All the above data suggested that under TCR stimulation the expression of miR-125a-5p could be upregulated via GATA3 in human nTreg cells.

### miR-125a-5p directly targets IL-6R and STAT3

**Figure 3:**
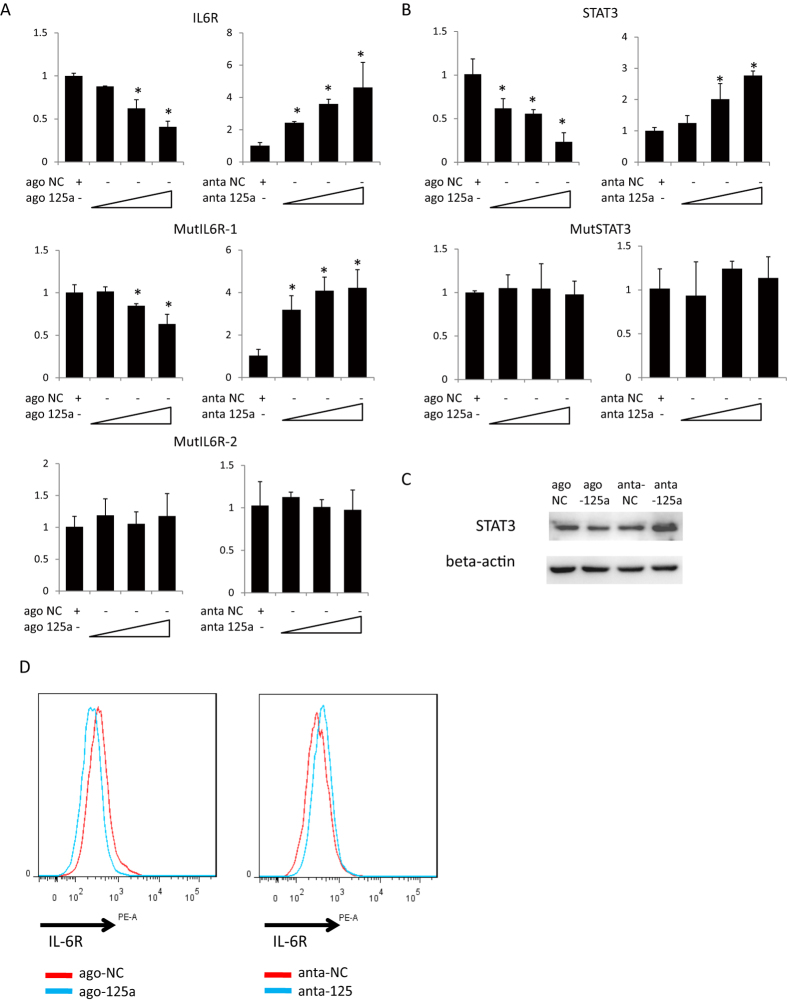
STAT3 and IL-6R are the targets of miR-125a-5p. (**A**) The luciferase activities of wild type or site directed mutation of the 3′UTR of IL-6R in 293T cells after overexpression or knock down of miR-125a-5p, *p < 0.01(t-test). (**B**) The luciferase activities of wild type or site directed mutation of the 3′UTR of STAT3 in 293T cells co-transfected with ago or anta miR-125a-5p. *p < 0.01(t-test). (**C**) Western blot of STAT3 in primary nTreg cells with differentially expressed miR-125a-5p. Data are representative of three independent experiments with similar results. All samples of cells were derived from the same experiment and the blots were processed in parallel. Full-length blots are presented in Supplementary Fig. 2. (**D**) Flow cytometry assay to detect protein level of IL-6R in primary nTreg cells after overexpression or knock down of miR-125a-5p. Data are representative of three independent experiments with similar results.

In order to investigate the function of miR-125a-5p we first identified its target genes. We searched for different target genes in TargetScan and then selected 42 target genes related to immune function. The 3′UTR of these genes were cloned into the pGL3 vector for luciferase assays. We overexpressed or knocked down miR-125a-5p in 293T cells and analyzed for changes in luciferase activity. The results show that miR-125a-5p significantly reduced luciferase activity using the 3′UTR of IL-6R and STAT3 but not after mutation of the miRNA interaction sites (MutIL6R-2 and MutSTAT3, respectively) (Fig. 3A,B). We then checked the protein levels of the target genes after overexpression or knockdown of miR-125a-5p in nTreg cells. Western blotting analysis showed that the protein level of STAT3 was reduced by miR-125a-5p (Fig. 3C & Supplemental Fig. 2) and by flow cytometry IL-6R was found decreased by miR-125a-5p (Fig. 3D). The miRNA binding sites and mutations are shown in Supplemental tables 1 and 2.

### MiR-125a-5p decreases the sensitivity of Treg cells toward IL-6 conversion

**Figure 4:**
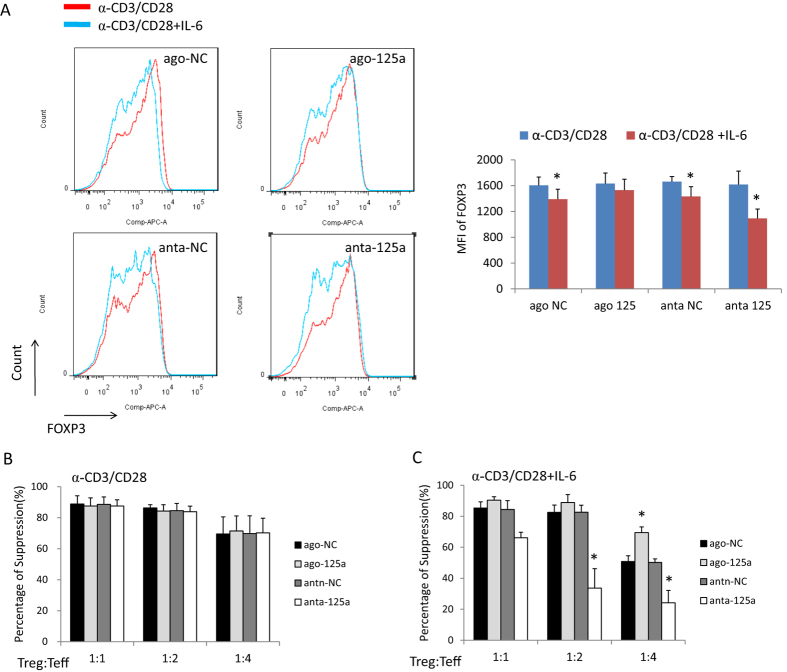
miR-125a-5p stabilizes nTreg cells towards IL-6 stimulation. (**A**) The flow cytometry data shows the change of expression levels of FOXP3 in primary nTreg cells after stimulation with IL-6. Flow cytometry plots (left) shows one representative experiment of three independent experiments with similar results and graph (right) shows MFI of FOXP3 from three individual experiments. *p < 0.05 (t-test). (**B**) The suppressive function of Treg cells after over-expression or knockdown of miR-125a-5p was detected by CFSE dilution method. The data shows the percentages of non-proliferating CD8^+^T cells. (**C**) After IL-6 stimulation, a suppression assay was used to detect the function of nTreg cells with different expression levels of miR-125a-5p.

It is well known that IL-6 stimulation can reduce FOXP3 levels and decrease the suppressive function of Treg cells, which is dependent on STAT3. As IL-6R and STAT3 are both targets of miR-125a-5p, we hypothesized that miR-125a-5p can influence the sensitivity of Treg cells towards IL-6 signals. After overexpressing or knockdown of miR-125a-5p in primary nTreg cells, the cells were activated with anti-CD3/CD28 beads with or without IL-6 for 48 hours. The expression level of FOXP3 in nTreg cells was then detected by flow cytometry. In the control groups (ago-NC and anti-NC), FOXP3 was downregulated when treated with IL-6. However, the expression of FOXP3 had almost no change upon IL-6 treatment in the miR-125a-5p overexpression group, and decreased significantly in the miR-125a-5p knockdown group (Fig. 4A). We also carried out a suppression assay with the same group of nTreg cells. The suppressive functions of the four different groups of Treg cells were similar when treated only with anti-CD3/CD28 beads (Fig. 4B). After IL-6 stimulation, the suppressive function of Treg cells decreased in all of the groups. Knockdown of miR-125a-5p highly sensitized the Treg cells to IL-6-mediated downregulation of Treg suppression, but the overexpression of miR-125a-5p increased the suppressive function of these Treg cells under IL-6 stimulation (Fig. 4C). All these data suggest that miR-125a-5p decreases the sensitivity of Treg cells toward IL-6-induced conversion.

### GATA3, miR-125a-5p, IL-6R, STAT3 and FOXP3 forms a regulatory loop

**Figure 5:**
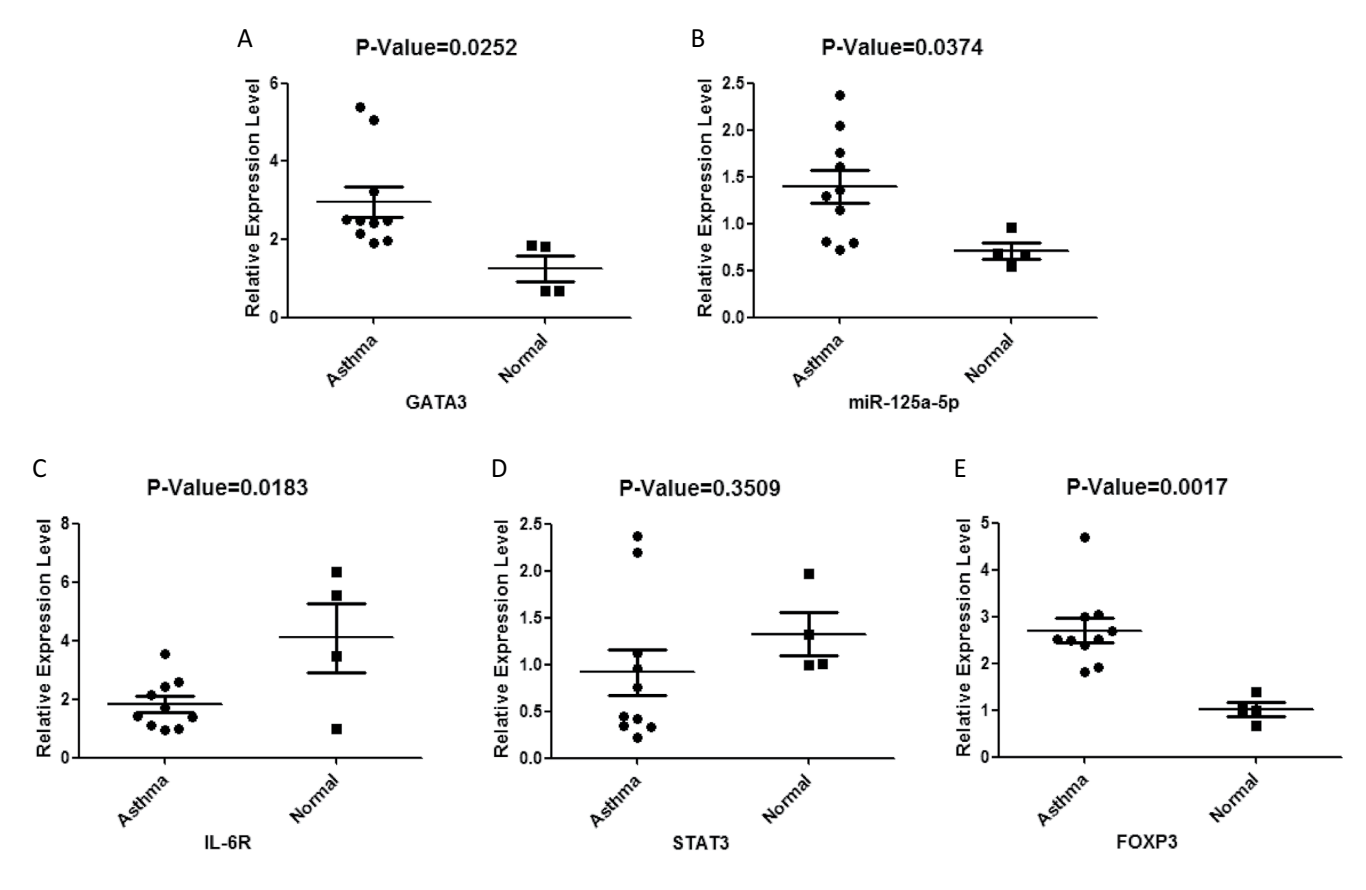
GATA3, miR-125a-5p, IL-6R and FOXP3 expression is perturbed in the Treg cells of asthma patients. The expression levels of GATA3 (**A**), miR-125a-5p (**B**), IL-6R (**C**), STAT3 (**D**) and FOXP3 (**E**) were detected in the Treg cells from asthma patients and healthy donors. The Treg cells were purified by FACS from PBMC.

As the decrease and dysfunction of Treg cells has been found in some human diseases we tested whether miR-125a-5p expression correlated with disease occurrence. Our published work has already shown how GATA3 expression is high in Treg cells of asthma patients^20^. Here, we investigated the expression level of miR-125a-5p and its target genes in asthma patients. The expression levels of GATA3 and miR-125a-5p were significantly upregulated in the Treg cells of asthma patients compared with healthy donors (Fig. 5A,B). In addition, the expression level of IL-6R was significantly downregulated in asthma patients (Fig. 5C). There was no difference in the expression of STAT3 between the asthma patients and healthy donors, albeit a cluster of patients did show relatively lower expression of STAT3 in the asthma patients (Fig. 5D). The expression of FOXP3 was also upregulated (Fig. 5E). These results reveal how miR-125a-5p and IL-6R may play important roles in asthma, and show a regulatory pathway that regulates the sensitivity of Treg cells toward IL-6 conversion.

## Discussion

We investigated the role of miR-125a-5p in human Treg cells. mir-125a-5p has been reported to act as a tumor suppression modulator in several kinds of cancers, such as glioblastoma, lung cancer, and hepatocellular carcinoma etc.^21^,^22^,^23^,^24^,^25^, and has also been reported to play a role in the immune system. In Drosophila, miR-125 was identified as a regulator of innate immunity^26^. In oxidized low-density lipoprotein-stimulated monocyte-derived macrophages, miR-125a-5p was found to decrease lipid uptake and secretion of inflammatory cytokines^27^. mir-125a-5p was also reported to downregulate the expression of a hepatitis B virus surface antigen^28^. During macrophage activation and polarization, miR-125a-5p promotes M2 macrophages and reduces M1 phenotype polarization^29^. However, the expression and function of miR-125a-5p in Treg cells remained unclear. The expression of miR-125a-5p is lower in CD4+ CD25+ T cells compared to CD4+ CD25− cells purified from human cord blood^30^. Valproate treatment of human cord blood CD4+ effector T cells could repress miR-125a-5p expression and upregulate FOXP3^31^. In purified T cell subsets from PBMC of human, Real-Time-PCR results indicated that the expression of miR-125a-5p is downregulated in Treg cells (resting) compared to effector T cells^32^. Our data also suggested that miR-125a-5p is expressed lower in FOXP3+ human T cell lines.

Many reports have indicated that miRNA expression can be regulated by transcription factors. The transcription factor Twist can induce miR-10b expression^33^ and HIF-1α can upregulate miR-210^34^,^35^. Our data showed that TCR and IL-2 activation could upregulate miR-125a-5p and this process was dependent on GATA3. GATA3 is the canonical transcription factor of Th2 cells^36^,^37^. GATA3 is required for T cell lineage commitment as GATA3 is expressed in Linloc-KithiCD25− early T cell progenitors and lack of GATA3 results in failed development of T cells^38^. GATA3 is indispensable for the early development of CD4+ T cells during the ß selection process^39^. It is reported that GATA3 binds to and promotes ThPOk to induce CD4+ and CD8+ T cell differentiation^40^. In Treg cells, the expression of GATA3 is critical for Treg cell physiology during inflammation^41^. GATA3 is also reported as essential for the function of Treg cells via binding to and promoting cis-acting elements of Foxp3^42^. We found that GATA3 upregulates miR-125a-5p in Treg cells during TCR stimulation. But in Th2 cells whether GATA3 can up regulate miR-125a-5p remains unknown. The expression of miR-125a-5p in resting Th1 and Th17 cells is higher than resting Treg cells, which may reflect other mechanisms that regulate miR-125a-5p in other T cell subsets.

In animals, miRNA sequences and their targets are not perfectly matched, which makes the search for targets complicated. Using a computer-based method, we identified IL-6R and STAT3 as two novel targets of miR-125a-5p. IL-6 transduces two signal pathways via binding to IL-6R: one dependent on STAT3 activation and another on Src homology region 2 domain-containing phosphatase 2 (SHP2). It is reported that the expression level of IL-6R is responsible for the sensitivity of Treg cells toward IL-6 conversion^43^. IL-6-induced inhibition of FOXP3 is dependent on STAT3^44^. Also, STAT3 has been reported to promote the instability of Treg cells^45^. Thus we predicted that miR-125a-5p could regulate the sensitivity of Treg cells under IL-6 stimulation and our *in vitro* data had confirmed our prediction. Our data also showed that without IL-6 stimulation, overexpression or knock down of miR-125a-5p did not change the expression level of FOXP3 or the suppressive function of Treg cells. Similar results have been reported that show how the overexpression of miR-125a have no effect on FOXP3 expression or cell phenotype^30^. Fayyad-Kazan H *et al.* indicated that valproate treatment induces FOXP3 expression in CD4+ effector T cells by increasing the binding of Ets-1 and Ets-2 to the FOXP3 promoter, instead of a miR-125a-5p dependent mechanism^31^. It is probable that different cell types and experimental conditions led to these different observations.

The IL-6 signal pathway is also important for the differentiation of iTreg and Th17 cells. However, changes in the expression level of miR-125a-5p in naïve T cells had no effect on the polarization of both cell types (data not shown). Also, the IL-6 signal pathway has been reported to be regulated by several miRNAs. In the malignant transformation of MCF-10A, Lin28 and let-7a regulate the activity of the IL-6/STAT3 axis^46^. It is reported that there is one feedback loop comprised of IL-6-STAT3-miR-24/miR-629-HNF4-miR-124, which regulates hepatocellular oncogenesis^47^. miR-93 influences proliferation and differentiation states of breast cancer stem cells (BCSCs) by targeting several genes including STAT3^48^. All these studies were performed in cancer cells and whether these miRNAs have similar roles to miR-125a-5p in Treg cells requires more investigation.

Asthma is a chronic inflammatory disease characterized by T helper cell 2 (Th2) inflammation leading to airway hyper-responsiveness (AHR). Evidence has indicated that Treg cells are involved in this disease^49^. Our data showed that GATA3, miR-125a-5p, FOXP3 and IL-6R are perturbed in the Treg cells of asthma patients. Further studies on functional and tissue-specific Treg cell subsets of asthma patients would be meaningful to reveal the functional consequences of altering this signal pathway.

In conclusion, we have identified a novel signal pathway in which miR-125a-5p decreases the sensitivity of Treg cells toward IL-6-mediated conversion. This signal pathway links two critical transcription factors in Treg cells with the function of one miRNA, which supports the notion that miRNAs are important regulators in Treg cells. Furthermore, miR-125a-5p and IL-6R are perturbed in asthma patients, which provides basis for development of new therapeutic strategies against asthma.

## Methods

### Cell culture and transfection

HEK293T cells were cultured in DMEM containing 10% fetal bovine serum (FBS) and transfected using Lipofectamine 2000 (Invitrogen) according to the manufacturer’s instructions. Jurkat and SZ4 cell lines were cultured in RPMI-1640 containing 10% FBS. Electroporation of Jurkat cells was performed with the NEPA21 apparatus (NEPAGENE, Japan).

### miRNA microarray

The microarray experiment was performed using the Agilent-021827 Human miRNA Microarray. The microarray data analyzed for this publication has been deposited in NCBI’s Gene Expression Omnibus and are accessible through GEO Series accession number; GSE64074 (http://www.ncbi.nlm.nih.gov/geo/query/acc.cgi?token=gjwremicrnkdjcl&acc=GSE64074)

### Real-time polymerase chain reaction assays

Total RNA was extracted using TRIzol reagent (Invitrogen). cDNA was synthesized using a reverse transcriptase kit (TaKaRa, Japan), followed by Real-Time-PCR analysis (SYBR Green; TaKaRa). The primers that were used are as follows:

ß-actin forward, 5′-GGACTTCGAGCAAGAGATGG-3′ and reverse, 5′-AGCACTGTGTTGGCGTACAG-3′; IL-6R forward, 5′-CCTGACGACAAAGGCTGTGCTCT-3′ and reverse, 5′-GCTGAACTTGCTCCCGACACTACTG-3′; STAT3 forward, 5′-GGGGCTTTTGTCAGCGATGGAGTA-3′and reverse, 5′-ATTTGTTGACGGGTCTGAAGTTGAG-3′; GATA3 forward, 5′- CTCATTAAGCCCAAGCGAAG-3′ and reverse, 5′- TTTTTCGGTTTCTGGTCTGG-3′; FOXP3 forward, 5′-TCCCAGAGTTCCTCCACAAC-3′ and reverse, 5′-ATTGAGTGTCCGCTGCTTCT-3′.

The expression level of miR-125a-5p was assayed with Hairpin-itTM MicroRNAs Quantitation PCR Kit (GenePharma, Shanghai, China). Each sample was analyzed in triplicate, and U6 RNA was used to normalize miRNA levels.

### Western blot analysis

Cells were washed with pre-chilled phosphate-buffered saline (PBS) and lysed in radioimmune precipitation assay buffer. These cell lysates were separated on SDS-PAGE gels, and transferred to PVDF membrane. All the primary antibodies were incubated overnight at 4 degrees Celsius, followed by incubation with HRP-conjugated goat anti-rabbit or goat anti-mouse secondary antibody and detected with ECL solution (Millipore). Antibodies against ß-actin were purchased from Sigma; anti-STAT3 (79D7) was purchased from Cell Signaling. Anti-GATA3 (HG3-31) antibody was purchased from Santa Cruz Biotechnology.

### Luciferase activity assay

The human 3′ UTR regions of IL-6R or STAT3 were amplified by PCR and cloned into the EcoRI and XhoI sites or KpnI and XbaI sites of the pGL3-control vector (Promega). The promoter region of miR-125a-5p was amplified by PCR and cloned into the KpnI and XhoI sites of the pGL3-basic vector (Promega). Nucleotide-substitution mutations were carried out using PCR-based methods. All primers used in vector construction are listed in the supplemental tables. All constructs were verified by sequencing. For the luciferase assay, 293T cells were cultured in 12-well plates, and transfected with 100 ng luciferase reporter plasmid, 5 ng pRL-TK vector expressing the Renilla luciferase (Promega), and 25nM, 50nM or 100 nM of ago miR-125a-5p or anta miR-125a-5p or miRNA negative control. For the promoter activity detection, 100 ng of luciferase reporter vector, 5 ng of pRL-TK vector and PIP-Flag-GATA3 or PIP-Flag-Blank vectors were co-transfected into 293T cells. After transfection for 48 hours, firefly and renilla luciferase activities were measured using the Dual-Luciferase Reporter Assay (Promega).

### Lentiviral constructs and infection

The shRNA lentiviral vectors pLKO.1 shGATA3-2 or pLKO.1 shCK were transfected into HEK 293T cells with the lentivirus packing vector Delta 8.9 and VSVG envelope glycoprotein. Viral supernatants were harvested after 48 h. Primary human Treg cells were transduced with virus along with a secondary anti-CD3/CD28 stimulus (four cells to one bead). The following shRNA sequence was used in this experiment: 5′-AGCCTAAACGCGATGGATATA-3′ (shGATA3-2).

### Human T cell culture

Naïve human CD4+ T cells (CD4+ CD25lowCD127highCD45RAhigh) and primary human CD4+ CD25highCD127low Treg cells from healthy donors were isolated by FACS on a BD FACS ARIA II sorter (BD Biosciences). Primary Treg cells were expanded using anti-CD3/CD28 dynabeads (Invitrogen) in X-VIVO-15 medium (Lonza, Switzerland) supplemented with 10% human AB serum, 1% GlutaMax (GIBCO), 1% sodium pyruvate (GIBCO), 1% Pen/Strep (GIBCO) and 100 U/ml IL-2. Naïve T cells were activated with anti-CD3/CD28 dynabeads in X-VIVO-15 medium and polarized into other T subsets under the following conditions: Th1: rhIL-12 (1 ng/ml) and anti-IL-4 (10 μg/ml) antibody; Th2: rhIL-4 (20 ng/ml) and anti-IFNγ (10 μg/ml) antibody; Th17: rhTGF-β1 (2.5 ng/ml), rhIL-6 (50 ng/ml), rhIL-1β (10 ng/ml), and rhIL-23 (100 ng/ml) and iTreg: 10 ng/ml TGF-ß, 100 ng/ml all-trans retinoic acid (atRA) and 100 units/ml rIL-2.

### Suppression assay

After culture for seven days, the Treg cells are restimulated with anti-CD3/CD28 dynabeads, 12.5 U/ml IL-2 and with or without 20 ng/ml IL-6 for two days. To measure suppression function, CFSE-labeled PBMC cells were stimulated with anti-CD3/CD28 dynabeads. To the responder cells, Tregs stimulated with or without IL-6 were added at different ratios and suppression of CFSE-labeled T cells was assessed as described^50^.

### Asthma Patients and Treg Isolation

The human patients and healthy control samples were from Ruijin Hospital (Shanghai, China). The study was approved by the Ruijin Hospital Ethics Committee, Shanghai Jiao Tong University School of Medicine (Permit number 2013-51) and was carried out in accordance with the approved guidelines. All participants provided written inform consent. The isolation of PBMC and Treg cells was performed as previously described^20^.

## Additional Information

**How to cite this article**: Li, D. *et al.* MiR-125a-5p Decreases the Sensitivity of Treg cells Toward IL-6-Mediated Conversion by Inhibiting IL-6R and STAT3 Expression. *Sci. Rep.* **5**, 14615; doi: 10.1038/srep14615 (2015).

## Supplementary Material

Supplementary Information
