# Supplementary material for: MiR-125a-5p Decreases the Sensitivity of Treg cells Toward IL-6-Mediated Conversion by Inhibiting IL-6R and STAT3 Expression
Source: Sci Rep. 2015 Oct 1;5:14615. doi: 10.1038/srep14615 (PMC4589732; doi:10.1038/srep14615)
Supplement: Supplementary Information [file srep14615-s1.pdf]

MiR-125a-5p Decreases the Sensitivity of Treg cells Toward IL-6-Mediated Conversion by Inhibiting IL-6R and STAT3 Expression

Dan Li[1,8*], Chao Kong[1,2,8], Andy Tsun[1], Chen Chen[1], Huihui Song[3], Guochao Shi[3],Wen Pan[4,5,7], Dai Dai[4,5], Nan Shen[4,5,6], and Bin Li[1*]

Fig S1.

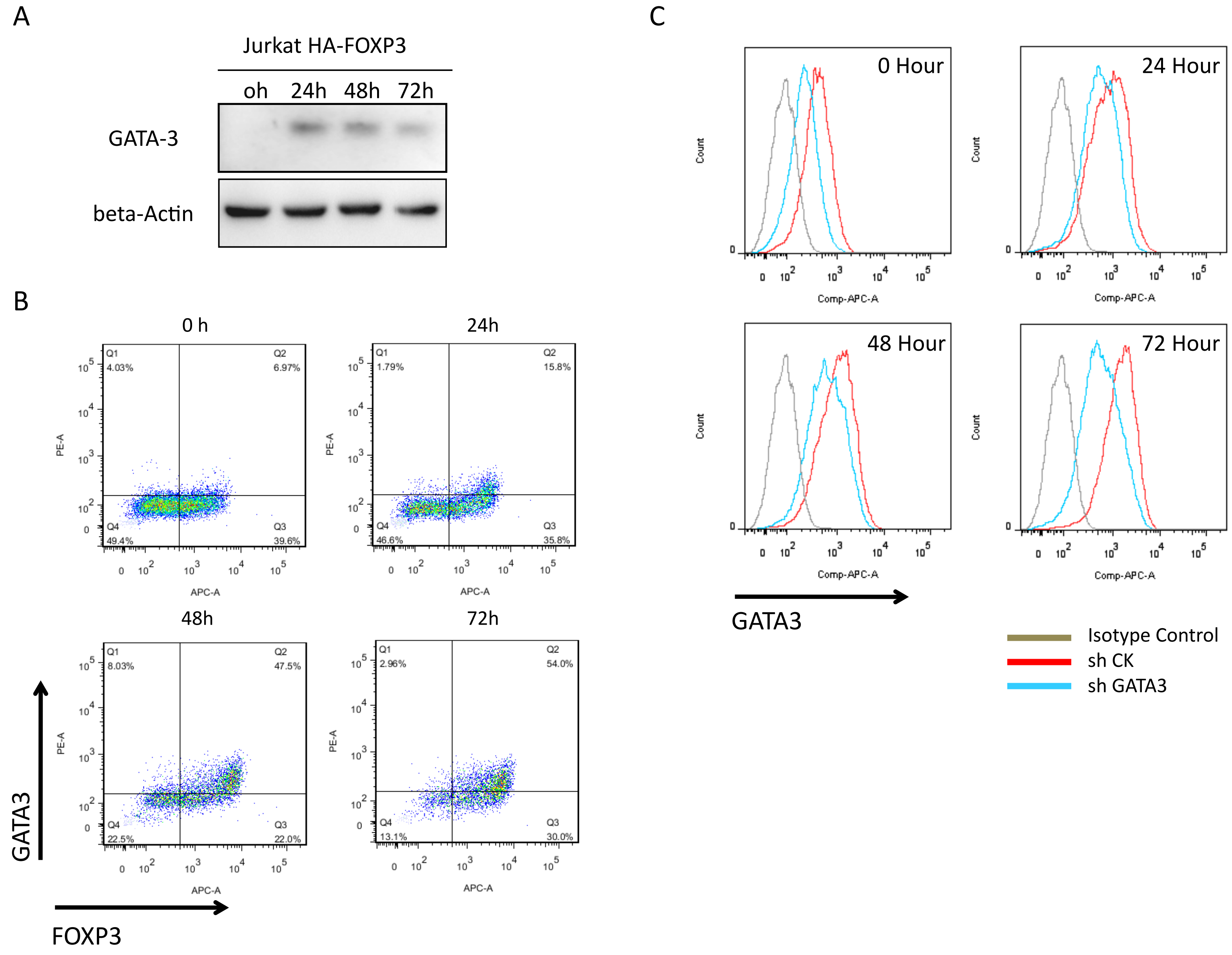

Fig S2.

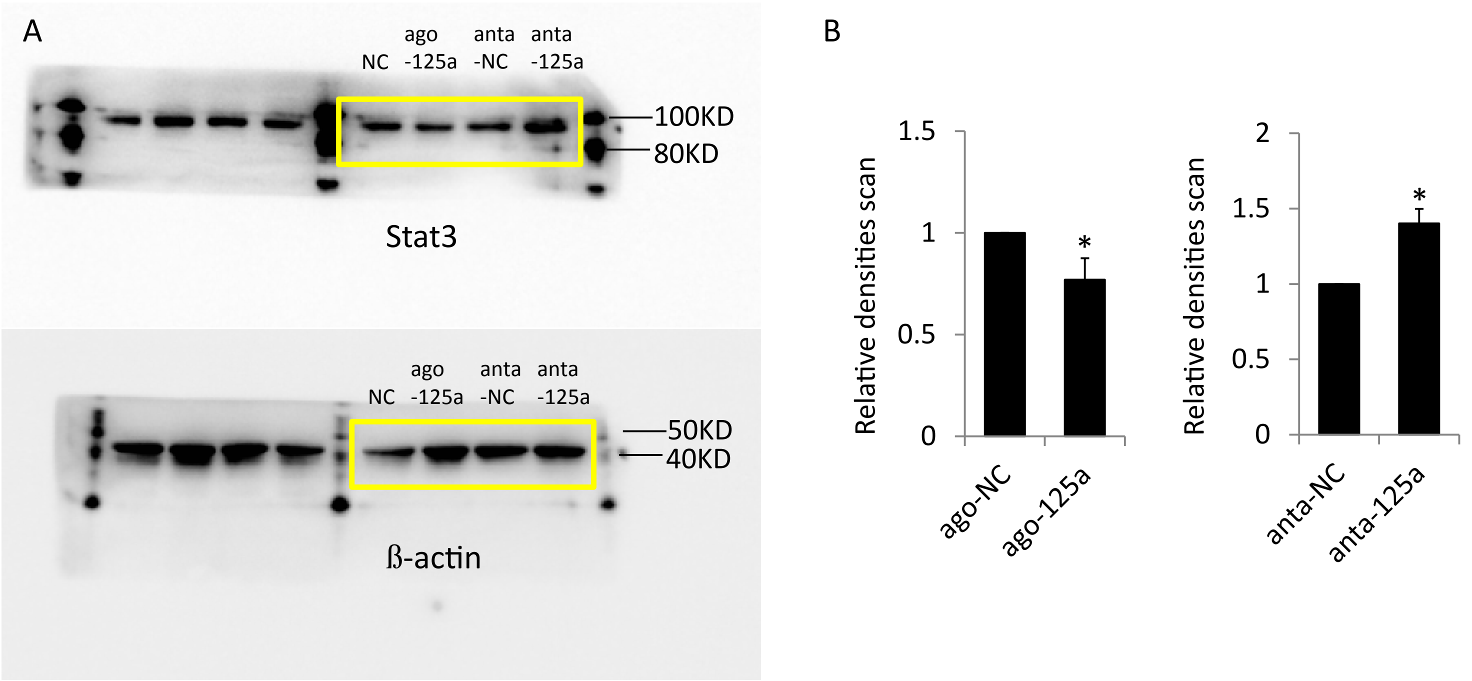
A
NC
ago -125a
anta -NC
anta -125a
100KD
80KD
Stat3
NC
ago -125a
anta -NC
anta -125a
50KD
40KD
ß-actin
B
Relative densities scan
1.5
1
0.5
0
*
ago-NC
ago-125a
Relative densities scan
2
1.5
1
0.5
0
*
anta-NC
anta-125a

Fig S1. GATA3 is induced by anti-CD3/CD28.

(A) The Western blot of GATA3 in Jurkat HA-FOXP3 after TCR stimulation at different time points.

(B) Flow cytometry analysis of GATA3 and FOXP3 in nTreg cells activated by anti-CD3/CD28 + IL-2.

(C) Flow cytometry analysis of GATA3 in GATA3 depleted or control nTreg cells after TCR stimulation.

Fig. S2. Full blots of figure 3C and relative densities scan.

(A)Western blotting data used anti-STAT3 and anti-ß-actin antibodies. All the samples of transfection cells derived from the same experiment and the blots were processed in parallel.

(B) The relative densities scan results of three replicates western blot.

Supplemental sequence 1.

The predicted binding sites of GATA3 in the promoter region of miR-125a-5p are underlined.

The promoter of miR-125a-5p

aggaagccaggttttctatcaggccatgccttccccaggaggcctgcctggccggagggaggaggggctgggggcagggactcct
gggtctctggggagaaggaggctttggatctggactcatgagtctgagggaggaaggggctgagtccttggattccaggaacatttgg
ggagaaaagggccagaggcctggattcctgggtctgtggtgaggaagggcctagacttctgggttctttagggaggaggggg**gatg**a
gagccttgactccagggtccctgatgaggaaggggctgagggcctggactcctgggttccttggggaggaggggccgggggcccg
gactcctgggtcctggcacccacccgtagaaccgaccttgcggggccttcgccgcacacaagctcgtgtctgtgggtccgtgtcggg
ggctcaccatcgcggctggggcctccccggccctccccctcatccctggtcctcctggtccctgtctgtctgtctgtcgggtctgtccac
ctgccgcgcccccgggctgag**gtag**gaggttgtatagttgaggaggacacccaaggagatcactatacggcctcctagctttcccc
aggctgcgccctgcacgggacggggcccggcggggacccccagccccactcagggacccttagccccactgggctgccccagg
gaccctgggaggaagagccgggctcttttctgtccttgtccctgcatcccctccttcccctgaaatctgttttccttccctgtctgtctc**catc**
tctgctgtgtctctgtggcttctgtgtctctttcacagtggatcctctgactccctcttattctggctttctaggtctctgcccctccc**gata**tct
ctctgtgtctctatttctgtcgtttttggtctttctgtctctggctctcagaatgtctctgtgc**ctat**ctccatctctgacccccaccccagggtct
accgggccaccgcacaccatgtTGCCAGTCTCTAGGTCCCTGAGACCCTTTAACCTGTGAGGA
CATCCAGGGTCACAGGTGAGGTTCTTGGGAGCCTGGCGTCTGGCC

Table S1

The binding sites of miR-125a-5p in the 3'UTR of IL-6R and STAT3

<table>
<tr><th>Site</th><th>Sequence</th></tr>
<tr><td>Position 621-627 of IL6R 3'UTR

hsa-miR-125a-5p</td><td>5' ...UGAAUAAUACAGUAUCUCAGGGC...
|||||||
3' AGUGUCCAAUUUCCCAGAGUCCCU</td></tr>
<tr><td>Position 93-99 of IL6R 3'UTR

hsa-miR-125a-5p</td><td>5' ...GCCAUGCCAGCUUAUCUCAGGGG...
|||||||
3' AGUGUCCAAUUUCCCAGAGUCCCU</td></tr>
<tr><td>Position 1532-1539 of STAT3 3' UTR

hsa-miR-125a-5p</td><td>5' ...ACGUGUCUGGUUGAGCUCAGGGA...
|||||||
3' AGUGUCCAAUUUCCCAGAGUCCCU</td></tr>
</table>

Table S2

The primer sequence for report vector construction.

Vector	Forward	Reverse
**pGL3-IL6R**	GAATTCTGACATCAGCAATACAGAC	CTCGAGACTCTACAGGGGATGGTA
**pGL3-STAT3**	GGTACCTACAGGTTGGACATGATGC	TCTAGACCAAGTCTCACCTTTCTAA
**pGL3-MutIL6R-1**	AGTCCCGTGTGCGGCCTTTGGCTTC	GATAAGCTGGCATGGCAGTGAGAAG
**pGL3-MutIL6R-2**	AGTCCCCCTGGTCGTTTTCAACAGAA	GATACTGTATTATTCACCCACTCAC
**pGL3-MutSTAT3**	AGTCCCAATATGGTTCTTAGCCAGTTTC	GCTCAACCAGACACGTCGCTGGGGC
**pGL3-pro125**	GGTACCTTCTATCAGGCCATGCCTT	CTCGAGCTCCCAAGAACCTCACCT
**Mut-1**	GTTCTTTAGGGAGGAGGGGAAAAAGAGCCTTGACTCCAGGG	CCCTGGAGTCAAGGCTCTTTTTCCCCTCCTCCCTAAAGAAC
**Mut-2**	CGCCCCCCGGGCTGAGAAAAGAGGTTGTATAGTTGAG	CTCAACTATACAACCTCTTTTCTCAGCCCGGGGGGCG
**Mut-3**	CCTTCCCTGTCTGTCTCAAAATCTGCTGTGTCTCTGTGG	CCACAGAGACACAGCAGATTTTGAGACAGACAGGGAAGG
**Mut-4**	GGTCTCTGCCCCTCCCAAAATCTCTCTGTGTCTC	GAGACACAGAGAGATTTTGGGAGGGGCAGAGACC
**Mut-5**	CTCAGAATGTCTCTGTGCAAAACTCCATCTCTGACCCCC	GGGGGTCAGAGATGGAGTTTTGCACAGAGACATTCTGAG
